# Supplementation with Fortified Lipid-Based and Blended Complementary Foods has Variable Impact on Body Composition Among Rural Bangladeshi Children: A Cluster-Randomized Controlled Trial

**DOI:** 10.1093/jn/nxaa061

**Published:** 2020-04-02

**Authors:** Saijuddin Shaikh, Rebecca K Campbell, Sucheta Mehra, Alamgir Kabir, Kerry J Schulze, Lee Wu, Hasmot Ali, Abu Ahmed Shamim, Keith P West, Parul Christian

**Affiliations:** The JiVitA Project of Johns Hopkins University, Bangladesh, Gaibandha, Bangladesh; Center for Human Nutrition, Department of International Health, Johns Hopkins University Bloomberg School of Public Health, Baltimore, MD, USA; Center for Human Nutrition, Department of International Health, Johns Hopkins University Bloomberg School of Public Health, Baltimore, MD, USA; Center for Human Nutrition, Department of International Health, Johns Hopkins University Bloomberg School of Public Health, Baltimore, MD, USA; International Centre for Diarrhoeal Disease Research, Bangladesh, Mohakhali, Dhaka, Bangladesh; Center for Human Nutrition, Department of International Health, Johns Hopkins University Bloomberg School of Public Health, Baltimore, MD, USA; Center for Human Nutrition, Department of International Health, Johns Hopkins University Bloomberg School of Public Health, Baltimore, MD, USA; The JiVitA Project of Johns Hopkins University, Bangladesh, Gaibandha, Bangladesh; Center for Human Nutrition, Department of International Health, Johns Hopkins University Bloomberg School of Public Health, Baltimore, MD, USA; James P Grant School of Public Health, Bangladesh Rural Advancement Committee (BRAC), University, Dhaka, Bangladesh; Center for Human Nutrition, Department of International Health, Johns Hopkins University Bloomberg School of Public Health, Baltimore, MD, USA; Center for Human Nutrition, Department of International Health, Johns Hopkins University Bloomberg School of Public Health, Baltimore, MD, USA

**Keywords:** childhood, complementary feeding, fat-free mass, fat mass, Bangladesh

## Abstract

**Background:**

Complementary food supplementation enhances linear growth and may affect body composition in children.

**Objective:**

We aimed to determine the effect of complementary food supplements provided from the age of 6 to 18 mo on fat-free mass (FFM) and fat mass (FM) gain among children in rural Bangladesh.

**Methods:**

In an unblinded, cluster-randomized, controlled trial we tested the effects of 4 complementary food supplements for 1 y [chickpea, rice lentil, Plumpy'doz, and wheat-soy-blend++ (WSB++)] compared with no supplements on linear growth. Body composition was estimated using weight-length-based, age- and sex-specific equations at 6, 9, 12, 15, and 18 mo and postintervention aged 24 mo. Generalized estimating equations (GEEs) were applied to estimate the effect of each complementary food on mean FFM and FM from 9 to 18 and 24 mo compared with the control, adjusting for baseline measures. Sex interactions were also explored.

**Results:**

In total, 3592 (65.9% of enrolled) children completed all anthropometric assessments. Estimated FFM and FM (mean ± SD) were 5.3 ± 0.6 kg and 1.4 ± 0.4 kg, respectively, at the age of 6 mo. Mean ± SE FFM and FM from 9 to 18 mo were 75.4 ± 14.0 g and 32.9 ± 7.1 g, and 61.0 ± 16.6 g and 30.0 ± 8.4 g, higher with Plumpy'doz and chickpea foods, respectively, than the control (*P* < 0.001). Estimated FFM was 41.5 ± 16.6 g higher in rice-lentil-fed versus control (*P* < 0.05) children. WSB++ had no impact on FFM or FM. A group-sex interaction (*P* < 0.1) was apparent with Plumpy'doz and rice-lentil foods, with girls involved in the intervention having higher estimated FFM and FM than control girls compared with no significant effect in boys. At 24 mo, FFM and FM remained higher only in girls eating Plumpy'doz compared with the controls (*P* < 0.01).

**Conclusions:**

In this randomized trial, supplementation effected small shifts in apparent body composition in rural Bangladeshi children. Where seen, FFM increments were twice that of FM, in proportion to these compartments, and more pronounced in girls. FFM increased in line with reported improvements in length. This trial was registered at clinicaltrials.gov as NCT01562379.

See corresponding commentary on page 1676.

## Introduction

An estimated 20% and 32% of children aged <5 y in low- and middle-income countries (LMICs) are underweight (weight-for-age *z* score <-2) and stunted (length-for-age *z* score <-2), respectively, with the prevalence highest in sub-Saharan Africa and South Asia (1). Growth deficits accrue relative to the WHO Growth Standard (2) between 6 and 24 mo, an age when human milk is gradually replaced as a primary food source, yet when children face limitations in the quality and quantity of complementary foods amidst high nutritional demands to support growth and development (3, 4).

Although anthropometric indicators are routinely measured to assess the growth impact of early childhood complementary feeding interventions, few studies partition weight change into body composition compartments of fat-free mass (FFM) and fat mass (FM). Where body composition has been examined, in relation to high-protein dietary intakes at different age groups in well-nourished populations in high-income countries, contradictory results have been found (5–8).

Numerous trials have been performed over the last decade to test small quantity lipid-based nutritional supplements (LNSs) to promote linear growth and reduce childhood stunting, underweight, wasting, and anemia, which was summarized in a recent Cochrane systematic review (9). Daily LNSs provided beyond the age of 6 mo through 18 or 24 mo significantly reduced the prevalence of moderate stunting by 7%, severe stunting by 15%, moderate wasting by 18%, moderate underweight by 15%, and anemia by 21%. A new meta-analysis, which included 18 LNS trials, found evidence of a 27% reduction in child mortality, highlighting the strong all-around benefit of this intervention in low-income settings (10). These LNS studies and systematic reviews have not reported body composition as an outcome although concerns have been raised that these supplements could lead to increased fatness (11, 12). In a cluster-randomized, controlled trial we conducted in rural Bangladesh, 4 complementary foods [chickpea, rice lentil, wheat-soy blend++ (WSB++), and Plumpy'doz] with nutritional counseling were provided from the age of 6 to 18 mo compared with nutritional counseling alone as a control (13). We found that the prevalence of stunting significantly declined by 5–6 percentage points in the Plumpy'doz and chickpea LNS versus control groups at the age of 18 mo. As a secondary outcome of that trial, we intended to explore the effect of the complementary food supplements on body composition. Thus, the objective of the present study was to determine the effect of providing daily complementary food supplements from 6 to 18 mo on changes in estimated FFM and FM for the duration of supplementation and again at age 24 mo (6 mo after feeding cessation) to examine the impact on long-term fat accretion with supplementation. In posthoc analyses we also explored these associations stratified by sex.

## Methods

### Population and study design

This trial was carried out in 19 Unions of Gaibandha and Rangpur districts in northwest Bangladesh under the “JiVitA” project implemented by Johns Hopkins University, USA. Briefly, this study was a community-based, unblinded, cluster-randomized (596 clusters) controlled trial of 4 complementary food supplements. This study assessed the effects of 3 specially formulated complementary food supplements and an international commercial product (Plumpy'doz) on child growth, with the group with no intervention intended as a negative control and the group receiving Plumpy'doz intended as a positive control in order to compare the growth of children given the other 3 food products. As a result of the multiple comparisons planned for the main outcome against these low and high controls, the number of cases to be assigned to negative and positive control groups was inflated by a factor of 1.7 relative to the other food interventions. The study design, sample size justification, allocation rates, randomization methodology, and complementary food supplement products have been previously described in detail (13). All infants identified by community surveillance were eligible and enrolled by the age of 6 mo from September 2012 to May 2013.

Children were assigned by area (study defined cluster) of residence to 1 of 5 groups: control (no food supplement), Plumpy'doz, an international, commercial, LNS (Nutriset, Malaunay, France), 2 locally developed lipid-based foods (1 rice and lentil based, and 1 chickpea based), and a World Food Programme (WFP)—designed fortified blended food called wheat-soy blend plus plus (WSB++). All supplements were provided weekly to children's homes for 1 y. For Plumpy'doz, 23 g/d (125 kcal) was provided for the first 6 mo (aged 6–12 mo) and 46 g/d (250 kcal) from the age of 13 to 18 mo. The locally developed rice-lentil and chickpea mixes, each provided in sachets of 23 g/d for daily use, were designed to be similar to the macro- and micronutrient composition of a conventional LNS (14), with both containing sugar, soybean oil, and whole milk powder. The composition of each food has been previously published (13) and is shown in **Supplemental Table 1**. For WSB++, a fortified blended flour, 32 g/d was cooked into porridge with water and served. Amounts of each supplemental food were designed to provide 125 kcal to infants aged 6–12 mo, with the daily serving doubled (rice lentil 56 g/d, chickpea 46 g/d, and WSB++ 64 g/d) to provide 250 kcal when children were aged 13–18 mo. Instructions for use were provided to caregivers, and intakes were monitored by field distributors twice a week. Nutritional counseling and age-specific messages on infant and young child feeding, health, and hygiene were provided by trained counselors to all 5 groups using modules developed by Alive and Thrive (15).

Written informed consent was obtained from all parents prior to participation. This study was approved by the Ethical Review Committee of the International Centre for Diarrhoeal Disease Research, Bangladesh (icddr, b) and the Institutional Review Board of the Johns Hopkins Bloomberg School of Public Health, MD, USA.

### Data collection

With birth dates known from an extant birth surveillance in the study area, 45 trained female interviewers visited families in their homes in the week the child became 6 mo old, informed parents/caregivers of the purpose and activities of the trial, obtained signed consent, and enrolled participants. Household socioeconomic data collected included parents’ education and occupation, monthly family income, and perceived household food security over the past 6 mo (16). Infant breastfeeding, complementary feeding history, and anthropometry were assessed at the age of 6, 9, 12, 15, 18, and 24 mo. All measurements were performed at home. Each anthropometrist was trained and standardized at the beginning of the study. The intra- and interworker technical errors of measurement (TEM) were calculated (17) and compared with cutoff points at 1.0% and 1.5% for intra- and inter-TEMs respectively. Workers whose calculated TEMs exceeded cutoffs during training were restandardized before conducting study measurements. Weight to the nearest 10 g was measured nude or with light clothing on a digital TANITA baby weighing scale (model BD585, Tanita Corporation of America), calibrated every morning using standard weights. Recumbent length was measured using a locally developed length board to the nearest 0.1 cm. Mid upper arm circumference (MUAC) was measured to the nearest 0.1 cm at the midpoint between the acromion and olecranon using a nonstretchable insertion measuring tape manufactured by JiVitA. Head and chest circumferences were measured to the nearest 0.1 cm using standard procedures with the same measuring tape. Length and circumferences were obtained in triplicate and the median value was used in the analysis.

Loss to follow-up including incomplete follow-up was ∼34% due to parents migrating from the study area to other places to earn money during the study period, anthropometry data missing related to noncooperation of children aged 18 and 24 mo. Only children who had completed all follow-up visits and had no missing anthropometry data were included in the analysis (66%).

In a planned subsample (*n* = 750, ∼14%), within a subset of 93 clusters centrally located in the study area and balanced by supplementation group, we conducted more intensive follow-up assessments including measurement of triceps and subscapular skinfold thicknesses and bioelectrical impedance at enrollment (6 mo), and aged 12 and 18 mo within 24 h of other anthropometric measurements. Triceps and subscapular skinfold thicknesses were measured to the nearest 0.2 mm using calipers (Holtain Ltd) and impedance at 5, 50, 100, and 200 kHz was measured using multifrequency bioelectrical impedance analysis (BIA) (model Quadscan 4000; Bodystat Ltd). Impedance at 50 kHz was used to estimate body composition in the subsample, which provided a basis for comparing and establishing the accuracy of Mellits–Cheek weight- and length-based equations used to estimate body composition in the full study cohort (18).

### Outcome variables

The primary outcome variable was body composition, partitioned into anthropometry-estimated FFM and FM compartments, determined in each child aged 6, 9, 12, 15, 18, and 24 mo. Body composition measures were expressed in units of weight (kg) or percentage of each component relative to total body weight. Measurements at the age of 6 mo provided the basis for presupplementation baseline estimates of body composition. Our primary interest was to assess the impact of the intervention over the time period from the age of 9 to 18 mo, with β-coefficients reflecting the mean annual rate of change in FFM and FM in an intervention group relative to the control group, assuming linearity. We believe this aggregate value to be a good indicator of the impact of the intervention as it captured growth trajectory for each child aged 9 to 18 mo. Additionally, we assessed body composition at 24 mo; that is, 6 mo after ceasing the feeding intervention trial.

### Body composition calculations

Body composition was estimated in the study using 3 different established equations: *1*) weight- and length-based equations (full trial cohort), *2*) skinfold- and MUAC-based equations (subsample), and *3*) BIA equations (subsample). Weight- and length-based equations for total body water (TBW, equations 1 and 2 below) were developed by Mellits and Cheek in 1970 for US boys and girls (18), and have been revalidated more recently against the deuterium (D_2_O) dilution method in Indian preschool children (19–21). FFM was derived using age- and sex-specific hydration factors of American reference children in the same age group (22). FM was estimated by the difference between body weight and FFM.

(1) \begin{eqnarray*} {\rm{Boys}}:\,{\rm{TBW}}\,\left( {{\rm{kg}}} \right)\,{\rm{ = - }}\,{\rm{1}}{\rm{.927 \, + }}\,{\rm{0}}{\rm{.465}}\,{\rm{ \times }}\,{\rm{W \, + }}\,{\rm{0}}{\rm{.045}}\,{\rm{ \times }}\,{\rm{H}}\\ \end{eqnarray*}

(2) \begin{eqnarray*} {\rm{Girls}}:\,{\rm{TBW}}\,\left( {{\rm{kg}}} \right)\,{\rm{ = }}\,{\rm{0}}{\rm{.076 \, + }}\,{\rm{0}}{\rm{.507}}\,{\rm{ \times }}\,{\rm{W}}\,{\rm{ + }}\,{\rm{0}}{\rm{.013}}\,{\rm{ \times }}\,{\rm{H}} \end{eqnarray*}

Where H = length in cm, W = weight in kg.

Skinfold- and MUAC-based equations developed by Shaikh and Mahalanabis (23) against the deuterium dilution method in Indian children were used to estimate FM (%) and FM (kg) in the subsample children. FFM was estimated by the difference between body weight (kg) and FM (kg). Equations 3 and 4 below were used to estimate percentage FM by means of tricipital and subscapular skinfolds, MUAC, and age in subsample children aged 6 (enrollment), 12, and 18 mo:

(3) \begin{eqnarray*} {\rm{Boys}}:\,{\rm{Fat}}\,\left( {\rm{\% }} \right)\, &=& \,{\rm{5}}{\rm{.304 + 0}}{\rm{.269}}\times \,{\rm{T + 0}}{\rm{.50}} \\ &&\times \,{\rm{S + 0}}{\rm{.685}}\, \times \,{\rm{M - 0}}{\rm{.063}}\, \times \,{\rm{A}} \end{eqnarray*}

(4) \begin{eqnarray*} {\rm{Girls}}:{\rm{Fat}}\left( {\rm{\% }} \right) &=& {\rm{7.017}}{\rm{- 0}}{\rm{.053 \times T + 0.201}}\\ &&\times \, {\rm{S + 0.765}}{\rm{\times M + 0}}{\rm{.052 \times A}} \end{eqnarray*}

Where T = triceps skinfold thickness in mm, S = subscapular skinfold thickness in mm, M = mid upper arm circumference in cm, and A = age in months.

In the subsample, impedance at 50 kHz was combined with anthropometry (length and weight) to provide an additional estimate of TBW using equation 5, which was then applied as above to calculate estimates of FFM and FM (24).

(5) \begin{eqnarray*} {\rm{TBW}}\,\left( {{\rm{kg}}} \right)\,{\rm{ = }}\,{\rm{0}}{\rm{.76 + 0}}{\rm{.18}}\,{\rm{ \times }}\,{{\rm{H}}^{\rm{2}}}{\rm{/}}{{\rm{Z}}_{{\rm{50}}}}{\rm{ \, + \, 0}}{\rm{.39}}\,{\rm{ \times }}\,{\rm{W}} \end{eqnarray*}

Where H = length in cm, W = weight in kg, Z_50_ = impedance at 50 kHz.

Correlation coefficients (r) were calculated for FFM and FM between the weight- and length-based body composition estimates and those obtained from the other approaches (skinfold- and MUAC-based equations and BIA) used in the subsample. Mean ± SD FFM, FM, and percentage body FFM and FM were also calculated at each time point using each technique. These approaches were used to validate and justify the use of the weight- and length-based equations to estimate body composition in the entire study cohort.

### Posthoc power calculation

Posthoc power calculations were performed for the primary outcome of aggregated FFM and FM. Power to estimate the detected difference found with Plumpy'doz compared with the control group was 76%, whereas detected differences with other supplements was lower at 10–50%.

### Statistical analysis

Household and maternal characteristics at enrollment and infant characteristics aged 6 mo (baseline) were expressed as *n* (%) or mean ± SD by intervention group to demonstrate comparability of the randomized groups. Similarly, comparisons were made using chi-square and *t* tests for discrete and continuous variables, respectively, between cases where children were in the current analysis and those excluded for reasons of missing data at 1 or more time points. Father's and mother's educational level was categorized as no education, completed class 1 to 5, completed class 6 to 9, and completed 10 or higher. Household monthly income from each family member including remittances was summed and classified as <50 US dollars (USD), 50–100 USD, and ≥101 USD. A sum of household food insecurity (HFI) scores was categorized at ≤9, 10–15, and ≥16.

We explored FFM and FM expressed as percent of total body weight by sex across all intervention groups to visualize sex-specific patterns in body composition by age in boys and girls. Aggregated data of total FFM and FM from the ages of 9 to 18 mo were normally distributed and therefore expressed as mean ± SD. In exploratory analyses, we compared total FFM and FM at each time point and the aggregated FFM or FM from 9 to 18 mo by intervention group within each sex using a 2 sample *t* test.

To estimate the effect of the interventions on the aggregated FFM and FM mean from each measure from 9 to 18 mo and separately at 24 mo, we used the generalized estimating equation (GEE) with identity link and exchangeable correlation to account for the cluster level correlation. The regression was the equivalent of a 1-factor ANOVA, with intervention groups represented by categorical variables and the counseling-alone control group as the reference. As body composition equations are sex specific, implying differential changes in body composition by sex, and because sex differences in body composition have been observed in other studies (25, 26), we tested interactions between child sex and the intervention groups on body composition in the GEE model. We set *P* < 0.10 as the significance level for interaction. As significant interactions were found, we present stratified analyses for boys and girls. Regression models were adjusted for the baseline measurement of FFM or FM.

Statistical significance level for all, but not the interaction, was set at *P* < 0.05. All statistical analyses were performed in STATA 11 (STATA Corp.) and SAS.

## Results

Parents of 5535 eligible children gave consent for participation, of which 98.4% (*n* = 5449) of children were enrolled. Anthropometry data were completed at all visits for 65.9% (*n* = 3592) of enrolled children (934 in the control group, 999 in Plumpy'doz, 549 in rice lentil, 546 in chickpea, and 564 in WSB++), who are included in this analysis (**Supplemental Figure 1**). On the other hand, *n* = 1857 children were missing at 1 or more visit (anthropometry = 937 and other information = 920). Analysis of baseline data reveal that children excluded from the analysis for reasons of incomplete follow-up visits were similar to children included in the analysis, although tended to come from better-off households ( **Supplemental Table 2**). Loss to follow-up between enrollment and 24 mo follow-up did not differ between groups.

The mean ± SD age of participants at enrollment was 6.1 ± 0.2 mo. Baseline characteristics of the enrolled children, their parents, and households were balanced across the randomized treatment groups (**Table 1**). Over 30% of fathers and ∼25% of mothers were uneducated, with nearly 15% of fathers and ∼10% of mothers educated beyond class 9. Most of the families had a monthly income of >100 USD and 52% of families had low HFI scores (9 or below). Mean ± SD of anthropometric and body composition measurements of participating children aged 6 mo are shown in Table 1 and did not differ by intervention group. However, across intervention groups, boys were consistently larger than girls in all anthropometric and body composition measures at the age of 6 mo, including weight (kg) (7.1 ± 0.9 compared with 6.5 ± 0.8, *P* < 0.0001), length (cm) (65.1 ± 2.4 compared with 63.3 ± 2.3, *P* < 0.0001), MUAC (cm) (13.6 ± 1.0 compared with 13.1 ± 1.0, *P* < 0.0001), head circumference (cm) (42.2 ± 1.3 compared with 41.1 ± 1.2, *P* < 0.0001), chest circumference (cm) (43.0 ± 2.1 compared with 41.7 ± 2.0, *P* < 0.0001), FFM (kg) (5.4 ± 0.6 compared with 5.3 ± 0.5, *P* < 0.0001), and FM (kg) (1.7 ± 0.3 compared with 1.2 ± 0.3, *P* < 0.0001).

**TABLE 1:** Socioeconomic, anthropometry, and body composition of children at enrollment (aged 6 mo) in 4 complementary food supplement groups and controls

Characteristics	Control (*n* = 934)	Plumpy'doz (*n* = 999)	Rice lentil (*n* = 549)	Chickpea (*n* = 546)	WSB++ (*n* = 564)
Father's education					
No schooling	356 (39.1)	355 (36.4)	198 (37.2)	191 (35.9)	208 (37.9)
Class 1 to 5	228 (25.0)	240 (24.6)	132 (24.8)	145 (27.3)	130 (23.7)
Class 6 to 9	204 (22.4)	225 (23.1)	133 (25.0)	115 (21.6)	132 (24.0)
Class 10 and above	123 (13.5)	155 (15.9)	69 (13.0)	81 (15.2)	79 (14.4)
Mother's education					
No schooling	238 (25.5)	236 (23.6)	125 (22.9)	132 (24.2)	127 (22.6)
Class 1 to 5	248 (26.6)	238 (23.8)	162 (29.6)	140 (25.6)	131 (23.3)
Class 6 to 9	360 (38.6)	421 (42.1)	214 (39.1)	217 (39.7)	243 (43.2)
Class 10 and above	86 (9.2)	104 (10.4)	46 (8.4)	57 (10.4)	62 (11.0)
Family income/month					
<50 USD	257 (28.0)	231 (23.3)	135 (25.1)	146 (27.1)	139 (25.0)
50–100 USD	342 (37.2)	353 (35.5)	193 (35.8)	186 (34.6)	185 (33.2)
≥101 USD	320 (34.8)	409 (41.2)	211 (39.1)	206 (38.3)	233 (41.8)
Household food insecurity					
HFI 9	467 (50.0)	519 (52.0)	303 (55.3)	290 (53.1)	289 (51.2)
HFI 10–15	346 (37.0)	363 (36.3)	190 (34.7)	186 (34.1)	202 (35.8)
HFI ≥16	121 (13.0)	117 (11.7)	55 (10.0)	70 (12.8)	73 (12.9)
Child parameters					
Weight, kg	6.8 ± 0.9	6.8 ± 0.9	6.8 ± 0.8	6.8 ± 0.9	6.7 ± 0.9
Length, cm	64.2 ± 2.5	64.2 ± 2.5	64.0 ± 2.6	64.3 ± 2.5	64.1 ± 2.6
MUAC, cm	13.3 ± 1.0	13.4 ± 1.1	13.3 ± 1.0	13.4 ± 1.0	13.4 ± 1.0
Head circumference, cm	41.6 ± 1.4	41.7 ± 1.3	41.6 ± 1.3	41.6 ± 1.4	41.6 ± 1.3
Chest circumference, cm	42.3 ± 2.2	42.4 ± 2.2	42.3 ± 2.0	42.4 ± 2.3	42.2 ± 2.0
Fat-free mass,^1^ kg	5.3 ± 0.6	5.3 ± 0.6	5.3 ± 0.5	5.3 ± 0.6	5.3 ± 0.6
Fat mass,^1^ kg	1.4 ± 0.4	1.5 ± 0.4	1.4 ± 0.3	1.4 ± 0.4	1.4 ± 0.3

HFI, household food insecurity; MUAC, mid upper arm circumference; USD, US dollars; WSB++, wheat-soy blend plus plus; USD, US dollars.

Variables are *n* (%) or mean ± SD. Difference between sum of *n* and total N represent missing values.

1 Equations 1 and 2 were used to estimate fat-free mass and fat mass (18).

As described, FFM and FM were derived from sex-specific equations using weight and length measures subsequent to validation of these estimates. In the validation substudy, estimated FFM (kg) and FM (kg) were comparable between weight- and length-based equations and skinfold- and MUAC-based equations, and weight- and length-based equations and the BIA equation overall and by sex at each age (**Supplemental Table 3**), justifying the use of the former to generate FFM and FM estimates for the full trial cohort. Correlation coefficients of FFM and FM between weight- and length-based equations and MUAC- and skinfold-based equations were 0.97–0.99 for FFM and 0.93–0.99 for FM. Associations were somewhat less strong between the BIA-based equation and weight- and length-based equations for FFM (0.94–0.98) and FM (0.85–0.96). Similar values in total and percent FFM and FM with age occurred regardless of method used (Supplemental Table 3).

**FIGURE 1:**
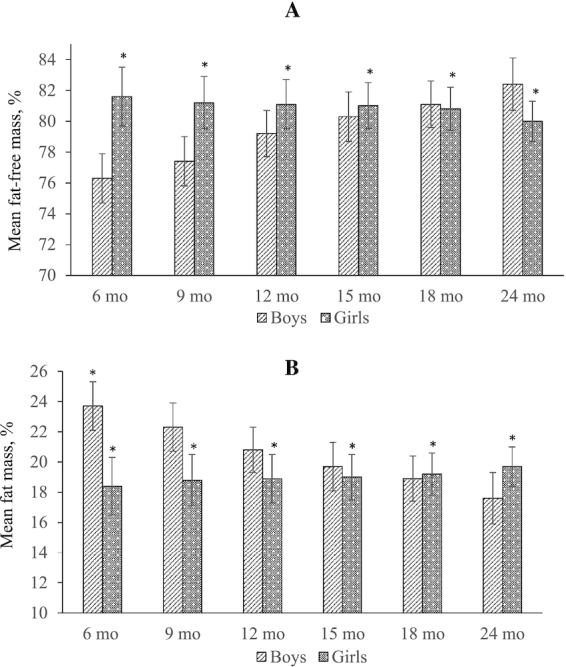
Relative fat-free mass (A) and fat mass (B) of children aged 6–24 mo in Bangladesh randomized to receive 1 of 4 complementary food supplements daily from the age of 6 to 18 mo and a corresponding control group. Values are mean ± SD percent of total body weight represented by each body composition compartment. *n* = 1793 boys and 1799 girls at each time point. *Different than boys, *P* < 0.001.

When weight- and length-based equations were used to assess body composition in the entire population, the mean FFM (%) was significantly higher among girls compared with boys at 6 mo, and FFM (%) increased with age in boys but remained more or less static in girls (**Figure 1**, Panel A). FFM (%) was significantly higher in girls than boys aged from 6 to 15 mo (*P* < 0.001), but aged 18 and 24 mo, FFM (%) was greater in boys compared with girls. The inverse trend was observed for the mean FM (%) among girls compared with boys (Figure 1, Panel B).

The body composition measurements at each age and aggregated from 9 to 18 mo compared across food groups are presented by sex in **Table 2**. FFM and FM were higher (*P* < 0.05) in the Plumpy'doz group at all age groups during supplementation and in the chickpea group at 15 and 18 mo (*P* < 0.05) compared with the control group in girls, but not boys (Table 2).

**TABLE 2:** Body composition of children at different measurement points in 4 complementary food supplement groups and controls and by sex^1^

		Control	Plumpy'doz	Rice lentil	Chickpea	WSB++
	Age, mo	*n* = 934	*n* = 999	*n* = 549	*n* = 546	*n* = 564
Overall	FFM, kg					
	6	5.33 ± 0.57	5.34 ± 0.59	5.31 ± 0.53	5.34 ± 0.60	5.31 ± 0.56
	9	5.87 ± 0.63	5.92 ± 0.62	5.87 ± 0.59	5.91 ± 0.65	5.86 ± 0.59
	12	6.27 ± 0.66	6.36 ± 0.65*	6.29 ± 0.64	6.34 ± 0.69	6.29 ± 0.62
	15	6.62 ± 0.70	6.72 ± 0.68**	6.65 ± 0.67	6.71 ± 0.72	6.64 ± 0.65
	18	7.02 ± 0.73	7.13 ± 0.72**	7.06 ± 0.72	7.10 ± 0.76	7.06 ± 0.69
	Mean, 9–18	6.45 ± 0.66	6.53 ± 0.65*	6.47 ± 0.64	6.52 ± 0.69	6.46 ± 0.62
	24	7.79 ± 0.82	7.86 ± 0.78	7.78 ± 0.81	7.83 ± 0.87	7.79 ± 0.78
	FM, kg					
	6	1.44 ± 0.38	1.46 ± 0.37	1.44 ± 0.35	1.44 ± 0.36	1.43 ± 0.35
	9	1.53 ± 0.36	1.57 ± 0.34	1.53 ± 0.33	1.55 ± 0.36	1.52 ± 0.33
	12	1.56 ± 0.33	1.61 ± 0.32*	1.57 ± 0.31	1.59 ± 0.33	1.57 ± 0.30
	15	1.59 ± 0.31	1.64 ± 0.30**	1.61 ± 0.30	1.63 ± 0.32	1.60 ± 0.29
	18	1.65 ± 0.29	1.71 ± 0.30**	1.68 ± 0.30	1.69 ± 0.32	1.66 ± 0.29
	Mean, 9–18	1.58 ± 0.30	1.63 ± 0.30**	1.60 ± 0.30	1.61 ± 0.32	1.59 ± 0.28
	24	1.80 ± 0.32	1.82 ± 0.32	1.79 ± 0.32	1.81 ± 0.36	1.79 ± 0.32
Boys	FFM, kg	*n* = 456	*n* = 515	*n* = 275	*n* = 268	*n* = 279
	6	5.44 ± 0.59	5.40 ± 0.63	5.37 ± 0.56	5.37 ± 0.63	5.37 ± 0.56
	9	6.05 ± 0.62	6.02 ± 0.66	6.02 ± 0.62	6.02 ± 0.67	6.00 ± 0.60
	12	6.50 ± 0.65	6.51 ± 0.69	6.50 ± 0.66	6.51 ± 0.71	6.46 ± 0.62
	15	6.89 ± 0.68	6.91 ± 0.71	6.91 ± 0.67	6.91 ± 0.73	6.84 ± 0.64
	18	7.33 ± 0.68	7.37 ± 0.74	7.35 ± 0.73	7.34 ± 0.76	7.30 ± 0.69
	Mean, 9–18	6.69 ± 0.63	6.70 ± 0.68	6.69 ± 0.65	6.70 ± 0.71	6.64 ± 0.62
	24	8.19 ± 0.74	8.16 ± 0.76	8.15 ± 0.80	8.15 ± 0.86	8.11 ± 0.73
	FM, kg					
	6	1.71 ± 0.28	1.70 ± 0.30	1.68 ± 0.26	1.67 ± 0.30	1.67 ± 0.26
	9	1.75 ± 0.30	1.75 ± 0.31	1.73 ± 0.28	1.73 ± 0.32	1.70 ± 0.28
	12	1.72 ± 0.29	1.73 ± 0.31	1.73 ± 0.29	1.72 ± 0.31	1.69 ± 0.28
	15	1.69 ± 0.29	1.72 ± 0.31	1.72 ± 0.29	1.70 ± 0.30	1.67 ± 0.28
	18	1.72 ± 0.28	1.74 ± 0.31	1.74 ± 0.31	1.72 ± 0.32	1.69 ± 0.29
	Mean, 9–18	1.72 ± 0.27	1.73 ± 0.29	1.73 ± 0.28	1.72 ± 0.30	1.69 ± 0.27
	24	1.78 ± 0.32	1.77 ± 0.32	1.76 ± 0.35	1.76 ± 0.36	1.72 ± 0.32
Girls	FFM, kg	*n* = 478	*n* = 484	*n* = 274	*n* = 278	*n* = 285
	6	5.23 ± 0.54	5.28 ± 0.53	5.25 ± 0.50	5.31 ± 0.57	5.25 ± 0.55
	9	5.70 ± 0.60	5.82 ± 0.55**	5.72 ± 0.52	5.81 ± 0.62	5.75 ± 0.56
	12	6.05 ± 0.61	6.19 ± 0.56**	6.08 ± 0.55	6.19 ± 0.63*	6.12 ± 0.58
	15	6.36 ± 0.62	6.52 ± 0.58**	6.40 ± 0.57	6.51 ± 0.66*	6.45 ± 0.60
	18	6.73 ± 0.64	6.89 ± 0.60**	6.77 ± 0.58	6.87 ± 0.69*	6.82 ± 0.61
	Mean, 9–18	6.21 ± 0.60	6.35 ± 0.56**	6.24 ± 0.54	6.34 ± 0.63*	6.29 ± 0.57
	24	7.41 ± 0.69	7.53 ± 0.66*	7.41 ± 0.63	7.52 ± 0.76	7.47 ± 0.69
	FM, kg					
	6	1.19 ± 0.26	1.21 ± 0.26	1.20 ± 0.24	1.22 ± 0.28	1.21 ± 0.27
	9	1.32 ± 0.29	1.38 ± 0.27**	1.33 ± 0.25	1.37 ± 0.30	1.35 ± 0.27
	12	1.41 ± 0.29	1.47 ± 0.27**	1.42 ± 0.26	1.47 ± 0.30	1.45 ± 0.28
	15	1.48 ± 0.29	1.56 ± 0.27**	1.50 ± 0.27	1.55 ± 0.31*	1.53 ± 0.28
	18	1.60 ± 0.29	1.67 ± 0.28**	1.61 ± 0.27	1.66 ± 0.32*	1.64 ± 0.28
	Mean, 9–18	1.45 ± 0.28	1.52 ± 0.26**	1.47 ± 0.25	1.51 ± 0.30*	1.49 ± 0.27
	24	1.81 ± 0.32	1.87 ± 0.31*	1.82 ± 0.29	1.86 ± 0.35	1.85 ± 0.32

FFM, fat-free mass; FM fat mass; WSB++, wheat-soy blend plus plus.

Data presented as mean ± SD.

1 Equations 1 and 2 were used to estimate fat-free mass and fat mass (18).

*P* values are from an ANOVA test.

Different from control, *P < 0.05, **P < 0.01.

The effects of each complementary food on mean FFM and FM aggregated from the age of 9 to 18 mo both unadjusted and adjusted for baseline FFM or FM are shown in **Table 3**. In adjusted analyses, FFM and FM were higher by 75.4 ± 14.0 g and 32.9 ± 7.1 g, respectively, in the Plumpy'doz group and by 61 ± 16.6 g and 30 ± 8.4 g, respectively, in the chickpea group compared with the control group (*P* < 0.001 for all). Gains in total FFM from the adjusted models were also significant for the rice-lentil food group and approached significance for the WSB++ group, whereas FM gains relative to the control group were not significant for those foods.

**TABLE 3:** Effect of 4 complementary food supplements on mean fat-free mass and fat mass from the ages of 9 to 18 mo compared with the control^1^

	Fat-free mass (g)				Fat mass (g)			
	Unadjusted		Adjusted^2^		Unadjusted		Adjusted^3^	
Study intervention	β ± SE	*P* value	β ± SE	*P* value	β ± SE	*P* value	β ± SE	*P* value
Overall								
Control (*n* = 934)	Ref	—	Ref	—	Ref	—	Ref	—
Plumpy'doz (*n* = 999)	88.3 ± 29.7	0.003	75.4 ± 14.0	<0.0001	48.3 ± 13.7	<0.0001	32.9 ± 7.1	<0.0001
Rice lentil (*n* = 549)	20.8 ± 35.1	0.55	41.5 ± 16.6	0.012	16.2 ± 16.1	0.32	13.8 ± 8.4	0.10
Chickpea (*n* = 546)	70.4 ± 35.1	0.045	61.0 ± 16.6	<0.0001	31.9 ± 16.2	0.049	30.0 ± 8.4	<0.0001
WSB++ (*n* = 564)	15.9 ± 34.8	0.65	30.5 ± 16.4	0.06	7.3 ± 16.0	0.65	11.0 ± 8.3	0.19
Boys (*n* = 1793)								
Control (*n* = 456)	Ref	—	Ref	—	Ref	—	Ref	—
Plumpy'doz (*n* = 515)	9.8 ± 42.4	0.88	42.9 ± 18.4	0.019	15.6 ± 18.2	0.39	22.5 ± 9.1	0.014
Rice lentil (*n* = 275)	−1.9 ± 50.3	0.97	64.3 ± 21.8	0.003	9.8 ± 21.6	0.65	27.4 ± 10.9	0.012
Chickpea (*n* = 268)	1.7 ± 50.7	0.97	68.4 ± 22.0	0.002	0.8 ± 21.7	0.97	31.4 ± 10.9	0.004
WSB++ (*n* = 279)	−51.8 ± 50.1	0.30	9.3 ± 21.7	0.67	−30.1 ± 21.5	0.16	2.5 ± 10.8	0.82
Girls (*n* = 1799)								
Control (*n* = 478)	Ref	—	Ref	—	Ref	—	Ref	—
Plumpy'doz (*n* = 484)	144.6 ± 37.3	<0.0001	96.4 ± 17.3^4^	<0.0001	68.0 ± 17.5	<0.0001	46.0 ± 8.4^5^	<0.0001
Rice lentil (*n* = 274)	31.3 ± 43.8	0.48	11.8 ± 20.3^6^	0.56	15.8 ± 20.5	0.44	3.1 ± 9.8^7^	0.75
Chickpea (*n* = 278)	134.2 ± 43.6	0.002	55.5 ± 20.3^8^	0.006	60.4 ± 20.4	0.003	27.5 ± 9.8^9^	0.005
WSB++ (*n* = 285)	75.9 ± 43.3	0.08	48.3 ± 20.1^10^	0.016	40.5 ± 20.3	0.046	22.7 ± 9.7^11^	0.019

Ref, reference; WSB++, wheat-soy-blend plus plus.

Values are β±SE.

1 Using generalized estimating equation linear regression analysis.

2 After adjusting for baseline fat-free mass.

3 After adjusting for baseline fat mass.

4 *P*-interaction = 0.036 for sex × Plumpy'doz.

5 *P*-interaction = 0.054 for sex × Plumpy'doz.

6 *P*-interaction = 0.082 for sex × rice lentil.

7 *P*-interaction = 0.096 for sex × rice lentil.

8 *P*-interaction = 0.654 for sex × chickpea.

9 *P*-interaction = 0.796 for sex × chickpea.

10 *P*-interaction = 0.183 for sex × WSB++.

11 *P*-interaction = 0.170 for sex × WSB++.

The impact of the Plumpy'doz intervention was greater in girls than boys whereas FFM was greater in boys compared with girls in the rice-lentil group (both *P*-interaction < 0.1). The effect of chickpea and WSB++ did not differ between boys and girls (*P*-interaction > 0.1).

No significant effect of the interventions on FFM and FM was observed at the age of 24 mo, 6 mo after the end of supplementation, in boys and girls combined (**Table 4**). However, in girls only, the mean ± SE FFM and FM was greater by 74.2 ± 28.1 g and 36.5 ± 13.6 g, respectively, in the Plumpy'doz group (*P* < 0.05) relative to the control group after adjusting for baseline measures.

**TABLE 4:** Effect of 4 complementary food supplements on fat-free mass and fat mass aged 24 mo (postintervention) compared with the control^1^

	Fat-free mass (g)				Fat mass (g)			
	Unadjusted		Adjusted^2^		Unadjusted		Adjusted^3^	
Study intervention	β ± SE	*P* value	β ± SE	*P* value	β ± SE	*P* value	β ± SE	*P* value
Overall								
Control (*n* = 934)	Ref	—	Ref	—	Ref	—	Ref	—
Plumpy'doz (*n* = 999)	63.8 ± 36.7	0.08	50.1 ± 23.9	0.037	23.1 ± 14.8	0.12	14.3 ± 13.2	0.28
Rice lentil (*n* = 549)	−8.4 ± 43.4	0.85	13.7 ± 28.3	0.63	−5.8 ± 17.5	0.74	−7.2 ± 15.6	0.64
Chickpea (*n* = 546)	40.0 ± 43.5	0.36	30.0 ± 28.3	0.29	17.4 ± 17.6	0.32	16.3 ± 15.7	0.30
WSB++ (*n* = 564)	−5.7 ± 43.0	0.89	9.9 ± 28.1	0.72	−9.7 ± 17.4	0.57	−7.5 ± 15.5	0.63
Boys (*n* = 1793)								
Control (*n* = 456)	Ref	—	Ref	—	Ref	—	Ref	—
Plumpy'doz (*n* = 515)	−34.4 ± 49.6	0.49	0.1 ± 29.8	0.99	−8.1 ± 21.2	0.70	−1.5 ± 15.1	0.92
Rice lentil (*n* = 275)	−42.8 ± 58.9	0.47	26.0 ± 35.4	0.46	−15.6 ± 25.1	0.53	1.0 ± 17.9	0.95
Chickpea (*n* = 268)	−41.2 ± 59.3	0.49	28.0 ± 35.7	0.43	−16.2 ± 25.4	0.52	12.7 ± 18.1	0.48
WSB++ (*n* = 279)	−88.2 ± 58.6	0.13	−24.8 ± 35.3	0.48	−54.2 ± 25.0	0.030	−23.4 ± 17.9	0.19
Girls (*n* = 1799)								
Control (*n* = 478)	Ref	—	Ref	—	Ref	—	Ref	—
Plumpy'doz (*n* = 484)	124.0 ± 44.2	0.005	74.2 ± 28.1^4^	0.008	58.3 ± 20.4	0.004	36.5 ± 13.6^5^	0.007
Rice lentil (*n* = 274)	6.2 ± 51.9	0.91	−14.0 ± 33.0^6^	0.67	4.9 ± 24.0	0.84	−7.7 ± 16.0^7^	0.63
Chickpea (*n* = 278)	114.3 ± 51.7	0.027	32.9 ± 32.9^8^	0.32	49.9 ± 23.9	0.037	17.1 ± 15.9^9^	0.28
WSB++ (*n* = 285)	65.1 ± 51.3	0.20	36.6 ± 32.6^10^	0.26	34.4 ± 23.7	0.15	16.7 ± 15.8^11^	0.29

Ref, reference; WSB++, wheat-soy-blend plus plus.

Values are β±SE.

1 Using the generalized estimating equation linear regression analysis.

2 After adjusting for baseline fat-free mass.

3 After adjusting for baseline fat mass.

4 *P*-interaction = 0.073 for sex × Plumpy'doz.

5 *P*-interaction = 0.057 for sex × Plumpy'doz.

6 *P*-interaction = 0.418 for sex × rice lentil.

7 *P*-interaction = 0.711 for sex × rice lentil.

8 *P*-interaction = 0.930 for sex × chickpea.

9 *P*-interaction = 0.846 for sex × chickpea.

10 *P*-interaction = 0.198 for sex × WSB++.

11 *P*-interaction = 0.098 for sex × WSB++.

## Discussion

In a randomized controlled trial of 4 different complementary food supplements provided for 1 y starting from the age of 6 mo, children receiving Plumpy'doz and chickpea foods had higher total FFM and FM relative to a control group. These results are consistent with findings of significant improvements in linear growth and a reduction in stunting seen in the same 2 groups previously (13). In sex-specific analyses, girls gained more FFM and FM from the Plumpy'doz supplemental food relative to the control than boys, whereas boys demonstrated gains in FFM and FM relative to the control with the rice-lentil supplemental food. In line with this impact on linear growth, here we found that the magnitude of increase in FFM was ∼2 times greater, but proportionate to the higher total FFM levels, than the increase in FM. The benefits of the intervention largely did not persist 6 mo after the intervention ended.

Data on LNS-based supplementation and body composition are scarce in low-income countries. An LNS trial in Burkina Faso among children aged 6–23 mo with moderate acute malnutrition found increases in weight, FFM, and FM using the deuterium dilution method compared with those receiving a corn-soy blend, although the increase in FM was not excessive (27). In a randomized trial, conjugated linoleic acid provided as a supplemental dietary fatty acid at the age of 7 mo resulted in a decrease in body fatness in children aged 6–10 y compared with the control group (28).

Previous studies on body composition changes with infant formula products or therapeutic foods for acute malnutrition may offer some insight into the effects of nutrient-dense products on early life body composition. A study was conducted by Mehta et al. (29) in the USA, where infants were introduced to commercial foods (cereals, fruits, and vegetables) and body composition determined by DXA. They reported that commercial foods from the ages of 6 to 12 mo did not alter body composition during the first year of life. A systematic review and meta-analysis by Gale et al. (30) included 15 studies reporting that formula-fed children had higher FFM (kg) throughout the first year of life than breastfed children. However, FM was lower in formula-fed children than breastfed children at the age of 6 mo, but this effect was no longer observed at the age of 12 mo. Body composition differences in formula-fed children compared with breastfed children are hypothesized to be due to the higher protein and energy content of formulas compared with breast milk (29). In Bangladesh, Kabir et al. (31) measured body composition in 35 malnourished children aged 24–59 mo before and after 3 wk of food supplementation with either a high-protein diet (15% of total energy as protein, *n* = 20 children) or a standard protein diet (7.5% of energy as protein, *n* = 15 children). The FFM over 3 wk in children fed a high-protein diet was higher than those who received the standard diet. Our results concur with these observations that FFM is more affected than FM by supplementation with energy- and nutrient-dense food.

At each age, FM (%) was lower in the study children compared with a group of American children, both in boys and girls (22). Even with a 4% increase in FM in the girls in our study, the FM and percent FM are lower than American children at each age group, suggesting little to no excessive increase in FM.

Differences in FFM and FM by sex in children has been reported by Shepherd et al. (32). Sex differences in absolute FM have also been reported in Bangladesh by Brown et al. (33) in a longitudinal growth study in a rural area and by West (34) in a nutritional survey in a semiurban area. We estimated percentage of mean FFM and FM from the age of 9 to 18 mo and found that FFM increased by ∼2% over this time period in both boys and girls. However, FM increased by 2% and 4% in boys and girls, respectively. FM has, in general, been shown to be higher in girls than boys from birth through childhood (22, 35–37). Although the observed 4% increase in FM in young girls in rural Bangladesh is twice that among boys, the percent body fat in this population remains less than reference American children (22). The difference in body composition between boys and girls might be due to sexual dimorphism that exists starting in utero and continues through childhood (25). Despite equal demands for energy in boys and girls during childhood, girls have greater FM after the age of 6 mo reflecting the very early preparation of the female body for future reproductive function (26). The biologic basis for a greater body composition response in girls to additional nutrients is not fully understood.

There were several strengths of the study. The body composition assessment method was validated with skinfold- and MUAC-based equations and the BIA-based equation in a subset of the population and agreement was excellent, allowing us to determine body composition estimates in a larger population than the validation subsample alone would allow. All anthropometric measures were obtained by extensively trained female interviewers. A potential limitation was that a more direct measure of body composition (e.g. deuterated water dilution or DXA method) was not done. However, more direct approaches are a challenge to implement in rural field settings. Analyses stratified by child sex were not specified a priori and we may not have enough power to detect effects for some comparisons. Despite this limitation, this study was able to detect evidence for concomitant gains in FFM and FM with the supplemental food intervention groups overall and in girls specifically in sex-stratified analyses relative to a control group that received information about child feeding alone.

In conclusion, 2 lipid-based complementary food supplements increased mean FFM and FM from the age of 9 to 18 mo suggesting that in addition to improving linear growth they also altered body composition, with some gains in the FFM compartment, a change that could reflect the promotion of skeletal growth. There was no evidence of excessive or exclusive increase in the FM compartment, which alleviates expressed concerns of the use of such interventions for reducing the global burden of undernutrition, especially stunting. In a secondary stratified analysis, there was some indication of a differential impact between boys and girls in some food groups. More research is needed into the mechanisms of sex differences in body composition. The lack of a sustained effect 6 mo postsupplementation suggests continued supplementation may be needed in this context with a high burden of food insecurity and growth faltering that continues through to the age of 2 y.

## Supplementary Material

nxaa061_Supplemental_File

## Notes

This research work was funded and supported by the USDA, National Institute of Food and Agriculture, under the Food and Nutrition Enhancement Program (Award No. 2010-38418-21732). DSM (Basel, Switzerland) provided micronutrient premix for the food supplements and Nutriset (Malaunay, France) provided Plumpy'doz. The Bill and Melinda Gates Foundation (GH 614) and the Johns Hopkins Sight and Life Global Nutrition Research Institute additionally supported this study.

Author disclosures: The authors report no conflicts of interest.

Supplemental Tables 1–3 and Supplemental Figure 1 are available from the “Supplementary data” link in the online posting of the article and from the same link in the online table of contents at https://academic.oup.com/jn/.

Abbreviations used: BIA, bioelectrical impedance analysis; FFM, fat-free mass; FM, fat mass; GEE, generalized estimating equation; HFI, household food insecurity; LNS, lipid-based nutrient supplement; MUAC, mid upper arm circumference; TBW, total body water; TEM, technical errors of measurement; USD, US dollars; WSB++, wheat-soy-blend plus plus.
