# Supplementary material for: Supplementation with Fortified Lipid-Based and Blended Complementary Foods has Variable Impact on Body Composition Among Rural Bangladeshi Children: A Cluster-Randomized Controlled Trial
Source: J Nutr. 2020 Apr 2;150(7):1924–32. doi: 10.1093/jn/nxaa061 (PMC7330466; doi:10.1093/jn/nxaa061)
Supplement: nxaa061_Supplemental_File [file nxaa061_supplemental_file.docx]

**Supplemental Table 1.** Nutrient intake recommendations and content of the supplementary food products per 100 g^1^

|  | Nutrient Content  (per 100 g of food) | | | | Daily Recommended Intakes | |
| --- | --- | --- | --- | --- | --- | --- |
|  | Plumpy’Doz | Rice Lentil | Chick pea | WSB ++ | 6-11 mo | 12-18 mo |
| Kcal | 532.33 | 500 | 520 | 382.3 | --- | --- |
| Protein, g | 12.72 | 8-10 | 10 | 19.3 | 11 | 13 |
| Fat, g | 34.48 | 32 | 31 | 10.1 | 30 | ND |
| Calcium, mg | 834.05 | 774 | 774 | 489.3 | 260 | 700 |
| Phosphorus, mg | 592.67 | 550 | 550 | 336.4 | 275 | 460 |
| Potassium, mg | 668.10 | 620 | 620 | 811.9 | 700 | 3000 |
| Zinc, mg | 8.62 | 8 | 8 | 7.6 | 7.6 | 3 |
| Copper, mg | 0.65 | 0.6 | 0.6 | 0.5 | 0.22 | 0.34 |
| Iron, mg | 19.40 | 18 | 18 | 9.2 | 11 | 7 |
| Iodine, µg | 118.53 | 110 | 110 | 56.9 | 130 | 90 |
| Selenium, µg | 36.64 | 34 | 34 | 42.8 | 20 | 20 |
| Magnesium, mg | 129.31 | 120 | 120 | 133.0 | 75 | 80 |
| Manganese, mg | 0.34 | 0.32 | 0.32 | 2.4 | 0.6 | 1.2 |
| Vitamin A, µg | 862.07 | 800 | 800 | 490.8 | 500 | 300 |
| Vitamin D, IU | - | 7.2 | 7.2 | 6.4 | 10 | 15 |
| Vitamin E, mg | 12.93 | 12 | 12 | 9.9 | 5 | 6 |
| Vitamin K, µg | - | 142 | 142 | 46.8 | 2.5 | 30 |
| Thiamine, mg | 1.08 | 1 | 1 | 0.6 | 0.3 | 0.5 |
| Riboflavin, mg | 1.08 | 1 | 1 | 0.8 | 0.4 | 0.5 |
| Niacin, mg | 12.93 | 12 | 12 | 11.3 | 4 | 6 |
| Folic acid, µg | 344.83 | 120 | 120 | 143.6 | 80 | 150 |
| Vitamin B12, µg | 1.94 | 1.8 | 1.8 | 2.3 | 0.5 | 0.9 |
| Vitamin B6, µg | 1.08 | 1 | 1 | 1.2 | 0.3 | 0.5 |
| Vitamin C, mg | 64.66 | 60 | 60 | 99.4 | 50 | 15 |
| Pantothenate, mg | 4.31 | 4 | 4 | 2.4 | 1.8 | 2 |

^1^Adapted from reference (13)

**Supplemental Table 2.** Comparison of household SES, child anthropometry and body composition at enrollment between analyzed and non-analyzed participants

| Characteristics | Analyzed group  n= 3592 | Non-analyzed group  n= 1802^1^ | P-value^2^ |
| --- | --- | --- | --- |
| **Father’s education**  No schooling  Class 1 to 5  Class 6 to 9  Class 10 and above | 1308 (37.4)  875 (25.0)  809 (23.1)  507 (14.5) | 655 (36.4)  422 (23.4)  390 (21.6)  335 (18.6) | 0.002 |
| **Mother’s education**  No schooling  Class 1 to 5  Class 6 to 9  Class 10 and above | 824 (23.6)  890 (25.5)  1436 (41.0)  347 (9.9) | 439 (24.4)  440 (24.4)  659 (36.6)  263 (14.6) | <0.0001 |
| **Household food insecurity**  HFI 9  HFI 10 – 15  HFI≥ 16 | 1837 (52.5)  1244 (35.6)  418 (11.9) | 882 (48.9)  659 (36.6)  261 (14.5) | 0.010 |
| **Child parameters** | | | |
| Weight (kg) | 6.77±0.88 | 6.82±0.94 | 0.055 |
| Length (cm) | 64.19±2.52 | 64.38±2.67 | 0.013 |
| MUAC (cm) | 13.36±1.03 | 13.41±1.08 | 0.12 |
| Head circumference (cm) | 41.62±1.35 | 41.72±1.40 | 0.014 |
| Chest circumference (cm) | 42.35±2.15 | 42.47±2.42 | 0.061 |
| Fat free mass (kg) | 5.32±0.57 | 5.36±0.61 | 0.018 |
| Fat mass (kg) | 1.44±0.36 | 1.46±0.37 | 0.15 |

HFI, household food insecurity; MUAC, mid upper arm circumference

Variables are n (%) or mean±SD

^1^Missing data 55

^2^From t-test for comparison between analyzed and non-analyzed participants

**Supplemental Table 3.** Mean±SD, percent and correlation coefficient (r) of fat free mass and fat mass measured by three methods in the subsample participants

| Age, mo | n |  | Fat free mass | | | | | Fat mass | | | | |
| --- | --- | --- | --- | --- | --- | --- | --- | --- | --- | --- | --- | --- |
| ***Overall*** |  |  | Weight-and length-based Method^1^ | MUAC and skinfold-based Method^2^ | BIA Method^3^ | Correlation (r)^4^ | Correlation (r)^5^ | Weight-and length-based Method^1^ | MUAC and skinfold-based Method^2^ | BIA Method^3^ | Correlation (r)^4^ | Correlation (r)^5^ |
| 6 | 632 | kg  % | 5.3±0.6  78.7 | 5.5±0.6  80.6 | 5.5±0.6  80.6 | 0.97 | 0.94 | 1.5±0.4  21.3 | 1.3±0.3  19.4 | 1.4±0.3  19.6 | 0.93 | 0.85 |
| 12 | 633 | kg  % | 6.3±0.7  80.0 | 6.4±0.7  81.0 | 6.3±0.7  79.3 | 0.99 | 0.98 | 1.6±0.3  20.0 | 1.5±0.3  19.0 | 1.7±0.4  20.6 | 0.94 | 0.92 |
| 18 | 621 | kg  % | 7.1±0.7  80.9 | 7.1±0.8  81.3 | 6.8±0.7  78.2 | 0.99 | 0.98 | 1.7±0.3  19.1 | 1.7±0.3  18.7 | 1.9±0.4  21.8 | 0.95 | 0.93 |
| ***Girls*** |  |  |  |  |  |  |  |  |  |  |  |  |
| 6 | 309 | kg  % | 5.3±0.5  81.5 | 5.3±0.6  81.6 | 5.2±0.5  81.6 | 0.99 | 0.98 | 1.2±0.3  18.5 | 1.2±0.2  18.4 | 1.3±0.3  19.2 | 0.98 | 0.96 |
| 12 | 309 | kg  % | 6.1±0.6  81.0 | 6.2±0.7  81.2 | 6.0±0.6  79.7 | 0.99 | 0.98 | 1.5±0.3  19.0 | 1.4±0.2  18.8 | 1.6±0.3  20.3 | 0.98 | 0.94 |
| 18 | 303 | kg  % | 6.8±0.6  80.8 | 6.8±0.7  81.0 | 6.6±0.6  78.4 | 0.99 | 0.98 | 1.6±0.3  19.2 | 1.6±0.2  19.9 | 1.8±0.4  21.6 | 0.99 | 0.94 |
| ***Boys*** |  |  |  |  |  |  |  |  |  |  |  |  |
| 6 | 323 | kg  % | 5.4±0.6  76.1 | 5.6±0.6  79.6 | 5.7±0.6  80.0 | 0.99 | 0.98 | 1.7±0.3  23.9 | 1.5±0.3  20.4 | 1.5±0.4  20.0 | 0.94 | 0.94 |
| 12 | 324 | kg  % | 6.5±0.7  79.1 | 6.6±0.7  80.8 | 6.5±0.6  79.1 | 0.99 | 0.98 | 1.7±0.3  20.9 | 1.6±0.3  19.2 | 1.7±0.4  20.9 | 0.93 | 0.92 |
| 18 | 318 | kg  % | 7.3±0.7  81.0 | 7.4±0.8  81.4 | 7.1±0.7  78.0 | 0.99 | 0.98 | 1.7±0.3  19.0 | 1.7±0.3  18.6 | 2.0±0.4  22.0 | 0.93 | 0.93 |

MUAC, mid upper arm circumference; BIA, bioelectrical impedance analysis

Data presented mean±SD and percent (%)

^1^Equations 1 and 2 were used to estimate fat free mass and fat mass based on length and weight (18)

^2^Equations 3 and 4 were used to estimate fat free mass and fat mass based on MUAC, skinfolds and age (23)

^3^Equation 5 was used to estimate fat free mass and fat mass based on BIA (24)

^4^Correlation coefficients of fat free mass and fat mass between weight- and length based and MUAC-, skinfolds- and age based equations

^5^Correlation coefficients of fat free mass and fat mass between weight- and length based and BIA based equations

All correlation coefficients (r) are statistically significant (P<0.0001)

**Supplemental Figure 1.** Study participants and anthropometry follow-up by intervention arm

Child died

<7mo n=2

Aged out

at 7 mo n=22

Child died

<7mo n=0

Aged out

at 7 mo n=13

Child died

<7mo n=3

Aged out

at 7 mo n=16

Child died

<7mo n=4

Aged out

at 7 mo n=13

Child died

<7mo n=0

Aged out

at 7 mo n=13

Not met/

no info n= 60

Refused n= 0

Not met/

no info n=85

Refused n=3

Not met/

no info n=122

Refused n=7

Not met/

no info n= 58

Refused n= 5

Not met/

no info n=61

Refused n=3

Total Children

n= 5939

Control

n= 1591

Plumpy’doz

n= 1599

Rice lentil

n= 901

Chickpea

n= 920

WSB++

n= 928

Consented

n= 1462

Consented n= 1511

Consented n= 838

Consented n= 860

Consented n= 864

Enrolled*

n= 1438

Enrolled*

n= 1492

Enrolled*

n= 825

Enrolled*

n= 843

Enrolled*

n= 851

Complete data available

n=934

Complete data available

n=999

Complete data available

n=549

Complete data available

n=546

Complete data available

n=564

Child died n=5

Not met/

No info n=129

Refused n=1

Moved n=2

Adopted n=0

Missing anthropometry data^+^ n=150

Child died n=5

Not met/

No info n=161

Refused n=1

Moved n=0

Adopted n= 0

Missing anthropometry data^+^ n=130

Child died n= 4

Not met/

No info n=117

Refused n=5

Moved n=0

Adopted n=0

Missing anthropometry data^+^ n=150

Child died n= 2

Not met/

No info n=221

Refused n=6

Moved n=0

Adopted n=1

Missing anthropometry data^+^ n= 263

Child died n= 3

Not met/

No info n=243

Refused n=13

Moved n=0

Adopted n=1

Missing anthropometry data^+^ n= 244

*Enrolled at 6 months of age; No info = no information available at time of visit; ^+^missing data on at least one occasion at the 9, 12, 18, and 24-month anthropometry measurements
